# A Manual of Syphilis and the Venereal Diseases

**Published:** 1900-11

**Authors:** 


A Manual of Syphilis and the Venereal Diseases. By James Nevins Hyde, A. M., M. D., Professor of Skin, Genito-urinary and Venereal Diseases, Rush Medical College, Chicago; etc, etc., and Frank Hugh Montgomery, M. D., Associate Professor of Skin, Genito-urinary and Venereal Diseases, Rush Medical College, Chicago, etc. Second edition revised and enlarged. 58 text illustrations and 19 full-page lithographic plates. Philadelphia. W. B. Saunders & Co. [Price, $4.00.]

The designating of this work as a manual is hardly correct; for while Saunders's Manuals have attained an unexcelled reputation, they are in the main looked upon and considered in the light of small, handy, little volumes, whereas Hyde and Montgomery is more. It is a comprehensive and fairly exhaustive work on syphilis and the venereal diseases, so it really is more than a manual—it is a work. Twice as large as the first edition, it shows revision and a great deal of work, being practically a new volume. The illustrating is far and away the most prominent addition,—that one can see it at a glance—and Saunders has shown a charming disregard for iron-bound tradition in this part of the work, which is but another evidence that a book bearing the Saunders' stamp is as nearly perfect as it is possible to make it. Many of the lithographic plates are those which were seen in the Handy Atlas series, in the Mracek volume on syphilis and venereal diseases, published in this country by Saunders. They are a distinct aid to the text, which explains most lucidly and in concise form the appearances and conditions met with in the affections considered.

The authors have practically rewritten the whole book, and that portion devoted to gonorrhea is in fact new. It has been brought directly up to date, and new treatments are accorded full space and consideration. Not only do they tell what they consider the best methods, but they exhibit all the others which have any standing.

Great care has been taken in the section on syphilis to take up each lesion and consider it, and then the special affections and complications are taken up, and their treatment most carefully discussed. The disorders which are not really venereal are given special attention. To many patients and some practitioners this will be a Godsend. Another department, too often slighted, is that concerning instrumentation of the urethra, and Hyde and Montgomery pay closest attention to it, advising and cautioning. There are in this section illustrations showing how to pass a sound. For the sake of the perfection of the book, these illustrations are necessary, and are, as they should be, photographic of the various movements; but, generally speaking, one cannot be taught to pass a sound by pictures however perfect. Dr. Agnew's advice was to "pass a sound on yourself, then try it on some one else."

If one were to be captious and overcritical, it might be said that the error which distinguishes all genito-urinary and venereal disease works was apparent in Hyde and Montgomery,—the slighting of gonorrhea in women. The effects of the diseases are far more reaching in women than in men, and the treatment is, of course, very different, yet mighty little space is, as a rule, given to the subject in any work which deals with venereal diseases. The claim might be made that it concerns gynecology and properly belongs in works devoted to that specialty. Probably; but the fact remains that it is a venereal disease and as such belongs in and should have extended consideration in books concerning genito-urinary and venereal diseases. The endless chain of pus tubes which adorns the records of the gynecologic wards of hospitals all over the country, is mutely eloquent of the lack of knowledge of proper treatment of gonorrhea in women.

Those pages which Hyde and Montgomery do devote to the subject are full of information and good advice.

The first edition of this work was valuable; this second edition is invaluable,—not to the specialist, but to the general practitioner. He will know a lot more after he has read it than he knows now, and the money he pays for it will be the best professional investment he ever made in his life. N. W. W.

